# *Pseudomonas aeruginosa* ExlA and *Serratia marcescens* ShlA trigger cadherin cleavage by promoting calcium influx and ADAM10 activation

**DOI:** 10.1371/journal.ppat.1006579

**Published:** 2017-08-23

**Authors:** Emeline Reboud, Stéphanie Bouillot, Sabine Patot, Benoît Béganton, Ina Attrée, Philippe Huber

**Affiliations:** 1 Université Grenoble Alpes, CNRS ERL5261, CEA BIG-BCI, INSERM UMR1036, Grenoble, France; 2 CIRI, Centre International de Recherche en Infectiologie, Inserm U1111, Université Lyon 1, Ecole Normale Supérieure de Lyon, CNRS UMR 5308, Lyon, France; Columbia University, UNITED STATES

## Abstract

Pore-forming toxins are potent virulence factors secreted by a large array of bacteria. Here, we deciphered the action of ExlA from *Pseudomonas aeruginosa* and ShlA from *Serratia marcescens* on host cell-cell junctions. ExlA and ShlA are two members of a unique family of pore-forming toxins secreted by a two-component secretion system. Bacteria secreting either toxin induced an ExlA- or ShlA-dependent rapid cleavage of E-cadherin and VE-cadherin in epithelial and endothelial cells, respectively. Cadherin proteolysis was executed by ADAM10, a host cell transmembrane metalloprotease. ADAM10 activation is controlled in the host cell by cytosolic Ca^2+^ concentration. We show that Ca^2+^ influx, induced by ExlA or ShlA pore formation in the plasma membrane, triggered ADAM10 activation, thereby leading to cadherin cleavage. Our data suggest that ADAM10 is not a cellular receptor for ExlA and ShlA, further confirming that ADAM10 activation occurred via Ca^2+^ signalling. In conclusion, ExlA- and ShlA-secreting bacteria subvert a regulation mechanism of ADAM10 to activate cadherin shedding, inducing intercellular junction rupture, cell rounding and loss of tissue barrier integrity.

## Author summary

Pore-forming toxins are the most widespread toxins delivered by pathogenic bacteria and are required for full virulence. Pore-forming toxins perforate membranes of host cells for intracellular delivery of bacterial factors, for bacterial escape from phagosomes or in order to kill cells. Loss of membrane integrity, especially the plasma membrane, has broad implications on cell and tissue physiology. Here, we show that two members of a unique family of pore-forming toxins, secreted by *Pseudomonas aeruginosa* and *Serratia marcescens*, have the capacity to disrupt cell-cell junctions of epithelial and endothelial cells, hence breaching two major tissue barriers.

## Introduction

Multicellular organisms have developed barriers to protect their internal body from microbial invasion. Mucosae constituted of single-layered epithelial cells are the favoured tissue barriers that bacteria may cross. Pathogenic bacteria have engineered different weapons to traverse these borders, either going through the cells (transcellular route) or at cell-cell junctions (paracellular route). Opportunistic pathogens, like *Pseudomonas aeruginosa* or *Serratia marcescens*, can only cross epithelial barriers when tissues are damaged or proliferate after physical trauma or biological insults. Once across the epithelial layer, bacteria may cross the endothelium, translocate into the vascular system and disseminate into the body.

Exolysin (ExlA hereafter) is a pore-forming toxin recently identified in a subset of strains from *Pseudomonas aeruginosa* species, generally called PA7-like strains, from the name of the first-identified strain of this category [1–11]. *ExlA* gene is located in the same locus as *exlB*, forming a Two-Partner Secretion System, in which ExlB is required for ExlA secretion in the extracellular milieu [12].

PA7-like strains have been isolated from patients with various infection types, such as acute or chronic pneumonia, urinary infections, burns, otitis, and recently from a patient suffering from hemorrhagic pneumonia. The PA7-like strains isolated so far do not possess a Type III secretion system (T3SS) and their virulence potential on cellular models is mainly correlated with the level of secreted ExlA [3, 13]. In the mouse lungs, ExlA-secreting strains induced major injuries of the alveolo-capillary barrier, leading to pulmonary hemorrhages and allowing bacterial dissemination in the body. Osmotic protection assays revealed that the inner diameter of the pore formed by ExlA in the host plasma membrane is approximately 1.6 nm, likely allowing the trafficking of small molecules[12]. Pore formation ultimately provokes cell death by plasma membrane rupture, as observed by microscopy, or monitored by lactate dehydrogenase (LDH) release and propidium iodide (PI) incorporation [3, 12].

Homologous ExlA proteins were identified in related *Pseudomonas* species, such as *P*. *protegens*, *P*. *entomophila* and *P*. *putida* [12]. Importantly, a 35% identity was found with ShlA pore-forming toxin from *Serratia marcescens* [3], which displays the same domain organization and a comparable size (172 kDa for ExlA and 165 kDa for ShlA), and shares a similar secretory pathway [14]. ShlA forms pores of 1–2 nm, is cytolytic for various cell types and induces hemorrhagic pneumonia in humans and in infected mouse lungs [15–17]. Hence, ShlA is related to ExlA in several structural and functional aspects. Other related toxins have been identified based on sequence homology and secretion pathway, including HpmA from *Proteus mirabilis*, HhdA from *Haemophilus ducreyi*, PhlA from *Photorhabdus luminescens*, EthA from *Edwardsiella tarda* and ChlA from *Chromobacterium violaceum* [14, 18–22], for which very little information is available. Altogether, these toxins constitute a unique family of pore-forming toxins, for which neither the 3-dimensional structure, nor the potential oligomerization and mechanisms of pore formation are known, and importantly, for which the mechanism of toxicity remains elusive.

Here, we show that ExlA and ShlA have the capacity to disrupt the cell-cell junctions of epithelial and endothelial cells, using an indirect mechanism. The pore formed by either toxin in the host cell triggers a Ca^2+^ influx, which activates A Disintegrin And Metalloproteinase domain-containing protein 10 (ADAM10), a transmembrane metalloprotease, whose natural substrates are transmembrane proteins, including some cadherins. ExlA- and ShlA-dependent ADAM10 activation rapidly leads to E- or VE-cadherin cleavage in epithelial or endothelial cells, respectively. As cadherins are major determinants of intercellular adhesion, cadherin cleavage induces cell-cell junction breakdown and loss of tissue integrity.

## Results

### ExlA-secreting strains induce E- and VE-cadherin cleavage

**Fig 1:**
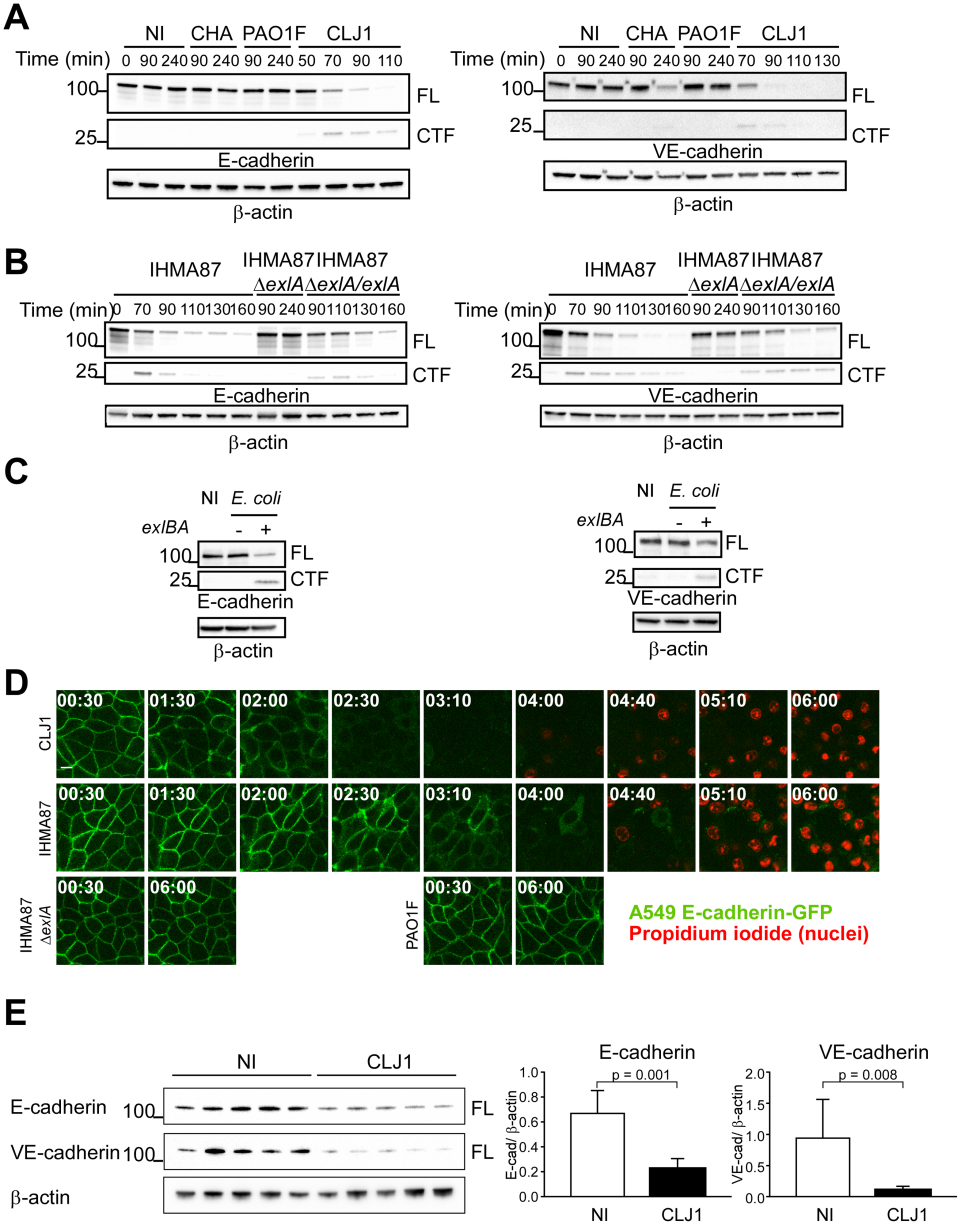
ExlA-dependent cleavage of E- and VE-cadherins. **A**. A549 cells (left) or HUVECs (right) were incubated with various *P*. *aeruginosa* strains: CHA, PAO1F or CLJ1, or were mock-infected with LB (NI). Cell extracts were prepared at different times post-infection, as indicated and analysed by Western blot using E-cadherin (left) or VE-cadherin (right) antibodies. In both cases, the full-length (FL) and post-cleavage C-terminal (CTF) fragments are shown. β-actin was used as loading control. The experiment was performed 3 times for A549 and twice for HUVECs with similar results. **B**. A549 cells (left) and HUVECs (right) were incubated with IHMA87, IHMA87Δ*exlA* or IHMA87Δ*exlA/exlA* strains, and cellular extracts were analysed as above. The experiment was performed 3 times for A549 and once for HUVECs. **C**. Similar experiments with A549 cells (left) or HUVECs (right) incubated with *E*. *coli* containing the empty vector (*exlBA* -) or *E-coli* expressing ExlB-ExlA *(exlBA +)*. **D**. A549-E-cadherin-GFP cells were incubated with CLJ1, IHMA87, IHMA87Δ*exlA* or PAO1F bacteria. E-cadherin-GFP (green) as well as nuclei labelling by propidium iodide (red) were followed by confocal videomicroscopy. Times post-infection are shown as “h:min”. One z-position is represented. The experiment was performed twice, with 4–5 positions recorded each time. All films showed similar results. **E**. Mice (5 per condition) were infected by bacteria inhalation (2.5x10^6^), using CLJ1 strain, or were mock-infected with PBS (NI). Mice were euthanized at 18 h.p.i.; protein extracts were prepared from lungs and were analysed by Western blot using E- and VE-cadherin antibodies (left). Histograms (right) show the E-cadherin/ β-actin and VE-cadherin/ β-actin ratios of band intensities, represented as means + s.d. Significance was calculated using Mann-Whitney’s test, as variances were not equal. The experiment was repeated once, with similar results.

We previously reported that T3SS-positive *P*. *aeruginosa* strains incubated with endothelial cells induce VE-cadherin cleavage, which is mediated by a protease (LasB) released by the T2SS [23]. LasB cleaves VE-cadherin in the middle of its extracellular domain, preventing its adhesive activity. We further showed that E-cadherin, located at epithelial cell-cell junctions, was resistant to LasB proteolytic activity [23]. As cadherins are required for tissue integrity, we tested the ExlA-secreting isolate CLJ1 for its capacity to cleave E-cadherin by incubation with the human alveolar cell line A549 (Fig 1A, left). CLJ1 induced a rapid and dramatic decrease in full-length E-cadherin levels, which paralleled the onset of a C-terminal E-cadherin fragment of 30 kDa. As previously reported [23], two ExlA-negative *P*. *aeruginosa* strains injecting ExoS, T and Y toxins through their T3SS, did not degrade E-cadherin, even at longer time points (Fig 1A, left). We also tested an ExlA-negative T3SS-positive strain injecting ExoU, a toxin endowed with phospholipase activity, known to induce membrane permeabilisation to nuclei dyes [24]. A549 cell incubation with the ExoU-positive strain did not induce E-cadherin cleavage (S1 Fig). Thus, membrane permeabilisation by a phospholipase is not sufficient to promote cadherin cleavage.

In similar experiments, VE-cadherin was rapidly degraded after incubation of primary human endothelial cells (HUVECs) with CLJ1 (Fig 1A, right). The onset of a C-terminal VE-cadherin fragment of 30 kDa was also observed concomitantly to the decrease of full-length VE-cadherin. As previously noticed, VE-cadherin was partially degraded due to LasB activity [23], however at much longer time points (Fig 1A, right).

To examine whether the rapid E- and VE-cadherin cleavage activity was dependent upon ExlA, we used the ExlA-secreting IHMA87 strain [12, 13] that can be manipulated genetically, as opposed to CLJ1. Hence, we tested the isogenic mutant IHMA87Δ*exlA* and its complemented counterpart IHMA87Δ*exlA/exlA* for their ability to degrade the cadherins (Fig 1B). IHMA87 similarly induced E-cadherin and VE-cadherin cleavages, albeit more slowly than CLJ1, while IHMA87Δ*exlA* did not, even at longer time points. The complemented strain recovered this ability. Thus, the cleavage of both types of cadherin is ExlA-dependent.

We previously reported that Type IV pili (“pili” hereafter) facilitated the ExlA-dependent toxicity of IHMA87 towards A549 cells, probably by enhancing bacterial adhesion [12]. We thus tested the effect of a mutant devoid of pili (IHMA87Δ*pilA*) on cadherin cleavage (S2 Fig). The lack of pili had no effect on E-cadherin cleavage and a very partial effect on VE-cadherin.

Next, we investigated whether other *P*. *aeruginosa* factors were needed for ExlA action on cadherins. As our attempts to purify a functional ExlA protein were unsuccessful, we used an *Escherichia coli* strain ectopically expressing ExlB-ExlA, and devoid of other *P*. *aeruginosa* factors. The *E*. *coli*-*exlBA* strain induced the cleavage of both E- and VE-cadherins, suggesting that ExlA alone can induce cadherin proteolysis (Fig 1C).

We then examined the fate of E-cadherin at cell-cell junctions by confocal videomicroscopy during infection using A549 expressing E-cadherin fused to GFP (Fig 1D). We observed a loss of E-cadherin-GFP labelling at cell-cell junctions when cells were incubated with CLJ1 or IHMA87, but not when incubated with IHMA87Δ*exlA* or PAO1F. This feature is in agreement with the above results by Western blot, except that the kinetics differ due to different experimental conditions. Taken together, the results show that ExlA-dependent E-cadherin cleavage promotes E-cadherin loss from cell-cell junctions. To assess cell membrane permeability, the non-permeant dye propidium iodide was added to the cell medium. Membrane disruption, as monitored by propidium iodide incorporation into cell nuclei, occurred much later than E-cadherin loss (Fig 1D). Thus, ExlA effects on E-cadherin could be observed earlier than those leading to cell death.

To definitely prove that ExlA induces barrier disruption, a bacterial transmigration assay was performed using Transwell filters. The bacterial transmigration across A549 monolayers were significantly decreased when IHMA87Δ*exlA* was used, compared to IHMA87 (S3 Fig).

To investigate the action of ExlA-secreting bacteria on cadherin cleavage in vivo, we infected mice by inhalation of a suspension of CLJ1 bacteria. IHMA87 was not used in this assay, as it is not a potent infective agent in this model [13]. Mice were euthanized at 18 h.p.i. and lung extracts were analysed by Western blot (Fig 1E). The data revealed that CLJ1-infected lungs contained significantly lower amounts of E- and VE-cadherins than the mock-infected lungs. Likewise, CLJ1 induces the cleavage of both cadherins in vivo. These results also show that ExlA can promote cadherin cleavage from a different species.

### ADAM10 is the executer of ExlA-dependent cadherin cleavage

**Fig 2:**
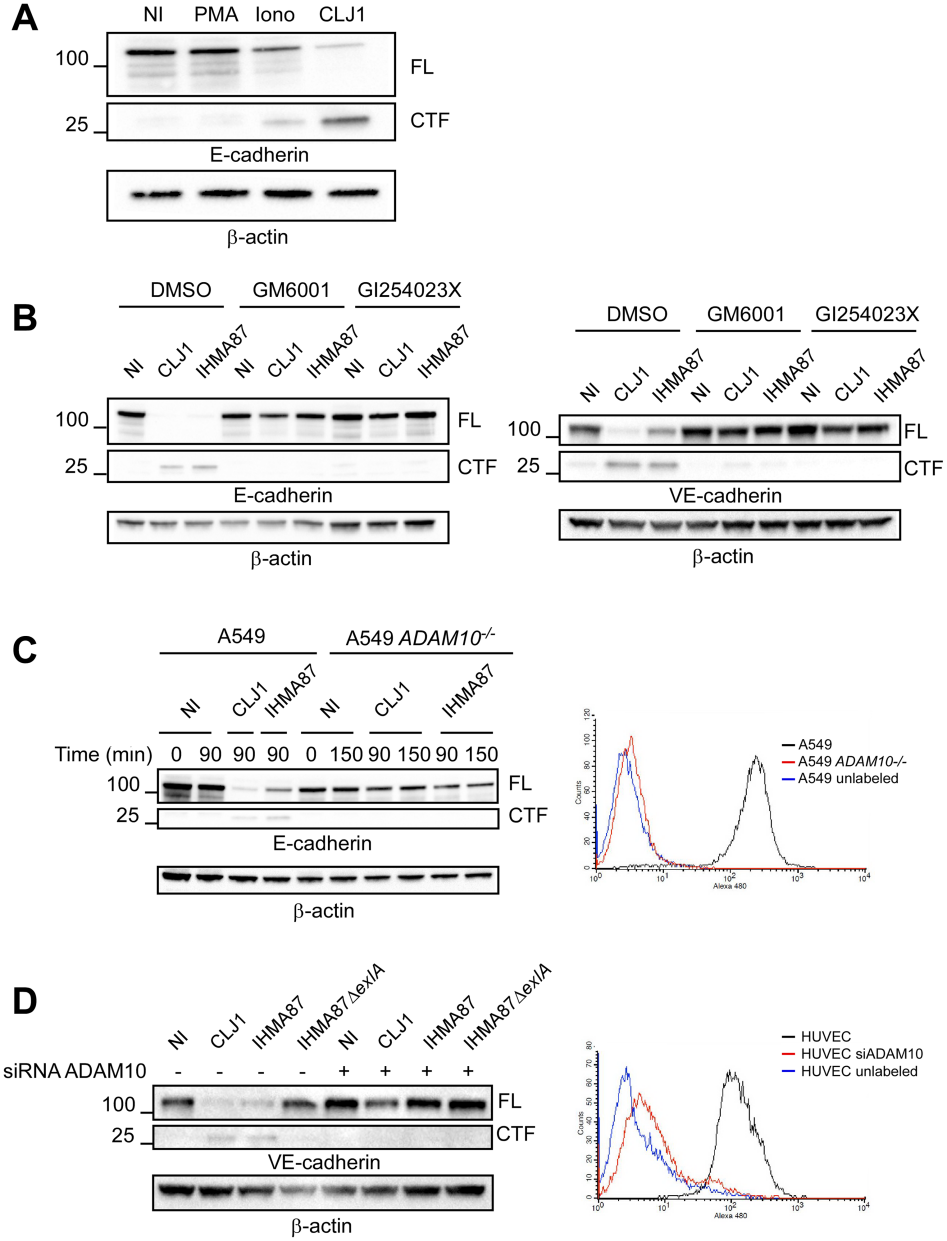
ADAM10 requirement for ExlA-dependent cadherin cleavage. **A**. A549 cells were either treated with 0.1 μg/mL PMA or 5 μM ionomycin (Iono) for 30 min, or infected with CLJ1 (90 min), or left untreated/uninfected (NI). Cellular extracts were analysed by Western blot using E-cadherin and β-actin antibodies. FL, full-length; CTF, C-terminal fragment. The experiment was performed twice. **B**. A549 cells (left) or HUVECs (right) were pre-treated with DMSO, the general metalloprotease inhibitor GM6001 (10 μg/mL) or the specific ADAM10 inhibitor GI254023X (5 μM) and then incubated with CLJ1 or IHMA87 (90 min), or uninfected (NI). Cellular extracts were analysed as above. The experiment was performed twice for A549 and 3 times for HUVECs. **C**. A549 or ADAM10-deficient A549 (A549 *ADAM10*^*-/-*^) cells were incubated with either CLJ1 or IHMA87. Cellular extracts were prepared at different time points post-infection as indicated and analysed by Western blot (left). The right panel shows the FACS analysis of ADAM10 surface expression of both cell lines, as well as the negative control. The experiment was performed 3 times. **D**. Similar experiment with HUVECs, either transfected with ADAM10 siRNA or untreated. The experiment was performed twice.

ADAMs are transmembrane metalloproteases, modulating cell-cell and cell-matrix interactions. They are major ectodomain sheddases, releasing a variety of cell-surface proteins. ADAM10 is an ubiquitous member of this family, for which several protein substrates have been identified, including E- and VE-cadherins [25–28]. ADAM10 cleaves both cadherins in their extracellular membrane-proximal domains, releasing the extracellular domain in the supernatant. The transmembrane domain is then rapidly cleaved by the γ-secretase and the cytoplasmic domain is targeted to the proteasome for degradation [26, 28]. In our experiments, the transient C-terminal cleavage fragment (30 kDa) induced by CLJ1 co-migrated with that produced by cell incubation with ionomycin, a potent inducer of ADAM10 activity [25, 29] (Fig 2A). ADAM17 activation by PMA did not produce a similar cleavage. Thus, these preliminary results suggested that ExlA could activate ADAM10, as has been reported for *Staphylococcus aureus* pore-forming toxin Hla [30, 31].

To further evaluate the role of ADAM10 in ExlA-dependent cadherin cleavage, we first used protease inhibitors: GM6001, a wide-spectrum Zn-metalloprotease inhibitor and GI254023X, a highly specific ADAM10 inhibitor [32]. Both inhibitors prevented E- and VE-cadherin cleavages after infection with CLJ1 or IHMA87 (Fig 2B). To definitely prove the role of ADAM10 in this mechanism, we generated ADAM10-deficient A549 cells, using CRISPR/Cas9 technology. The absence of ADAM10 was verified by FACS analysis (Fig 2C, right). ADAM10-deficient A549 cells were resistant to E-cadherin cleavage after incubation with CLJ1 or IHMA87 (Fig 2C, left). To evaluate the VE-cadherin cleavage in primary cells, we knocked down ADAM10 in HUVECs using siRNA, as it is not possible to generate gene deficiency in primary cells using the CRISPR/Cas9 system. ADAM10-surface expression was dramatically decreased in ADAM10 siRNA-treated HUVECs (Fig 2D, right). VE-cadherin cleavage was prevented in ADAM10 siRNA-treated HUVECs infected with CLJ1 and IHMA87 (Fig 2D, left).

Altogether, these findings demonstrate that ADAM10 is the executer of ExlA for cadherin cleavage and is absolutely required for this activity.

### ADAM10 is dispensable for the necrotic activity of ExlA

**Fig 3:**
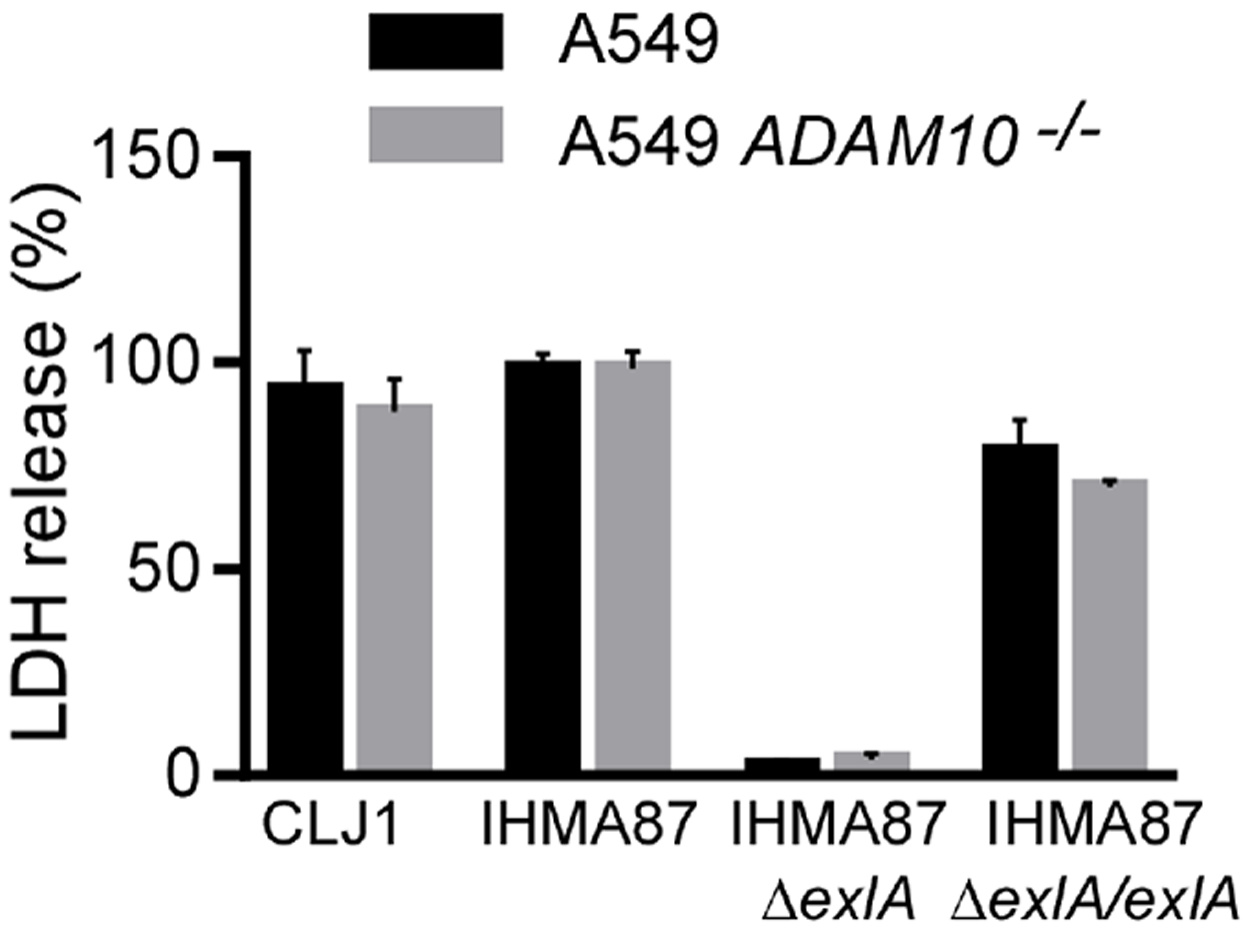
ExlA necrotizing activity is preserved in ADAM10-deficient cells. Plasma membrane rupture was monitored by LDH release in the supernatant. A549 or A549*ADAM10*^*-/-*^ cells were incubated for 5 hours with IHMA87, IHMA87Δ*exlA* or IHMA87Δ*exlA/exlA* strains. The supernatants were the tested for LDH activity. The histograms show the mean ± s.d. of triplicates. The data are representative of 3 experiments.

To examine whether ADAM10 was required for ExlA-dependent plasma membrane rupture, we tested the necrotic activity of ExlA in ADAM10-deficient A549 cells (Fig 3). LDH release was independent of the presence of ADAM10, showing that ADAM10 is not involved in cell death and that ADAM10 is not the membrane receptor of ExlA.

### ExlA triggers intracellular calcium elevation

**Fig 4:**
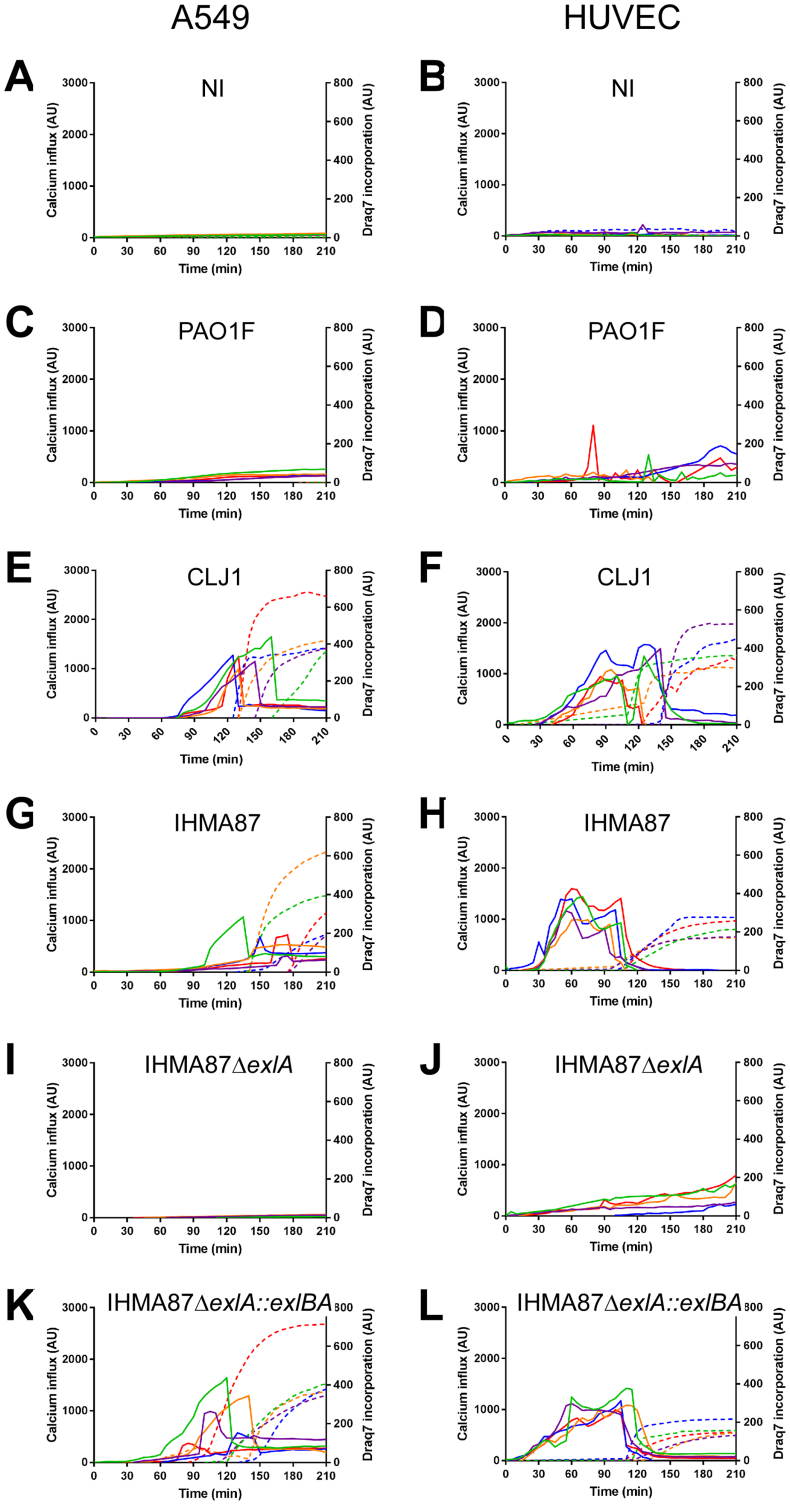
Intracellular calcium elevation generated by ExlA-secreting bacteria. Intracellular Ca^2+^ content was analysed by videomicroscopy using the permeant Fluo3-AM fluorescent probe. Plasma membrane rupture was evaluated using the non-permeant Draq7 fluorescent probe. A549 cells (**A,C,E,G,I,K**) or HUVECs (**B,D,F,H,J,L**) were either non-infected (**A,B**) or infected with PAO1F (**C,D**), CLJ1 (**E,F**), IHMA87 (**G,H**), IHMA87Δ*exlA* (**I,J**) or IHMA87Δ*exlA*::*exlBA* (**K,L**). The fluorescence intensities of five cells were analysed in each case; the Fluo3 intensities are represented by solid lines and the Draq7 intensities by dashed lines, using the same colour code for one cell. The data are representative of 4–7 experiments. Uninfected conditions were performed in each experiment.

As previously described, high cytosolic Ca^2+^ concentrations induce cadherin cleavage through activation of ADAM10 [26, 33], which was further confirmed here using the calcium ionophore ionomycin (Fig 2A). Therefore, we tested the capacity of ExlA-secreting strains to induce intracellular Ca^2+^ elevation in A549 cells and HUVECs. We used Fluo3-AM, a cell-permeant fluorescent probe, to monitor intracellular Ca^2+^ together with Draq7, a non-permeant fluorescent probe, to visualize necrotic cells after plasma membrane rupture. Cell fluorescence were recorded on both channels by videomicroscopy and their intensities measured on individual cells. No Fluo3 signal was obtained in uninfected conditions and inconsistent signals were detected when cells were infected with PAO1F (Fig 4A–4D), as previously reported [34]. In contrast, sharp increases of Fluo3 intensities were observed when either cell types were infected with CLJ1 (Fig 4E and 4F); this increase takes place earlier in HUVECs. Similar observations were made with IHMA87 infection (Fig 4G and 4H), except that Ca^2+^ rise was not consistent for each A549 cell and less marked in general. When IHMA87Δ*exlA* was used, no Ca^2+^ elevation was noted (Fig 4I and 4J), while Fluo3 signal increases were observable within IHMA87Δ*exlA*::*exlBA*-infected cells (Fig 4K and 4L). Hence, ExlA triggers a massive Ca^2+^ elevation in host cells, because of Ca^2+^ entry through the pore formed in the plasma membrane. ADAM10 may thus be activated by this process.

The increase of Fluo3 intensity was consistently followed by a sharp drop of fluorescence that was concomitant to Draq7 incorporation into the cells (Fig 4E–4H, 4K and 4L), suggesting that the Fluo3 diffused in the extracellular milieu when the plasma membrane was ruptured.

### ExlA-dependent calcium influx drives both ADAM10 activation and necrosis

The inactive form of ADAM10 (pro-ADAM10), linked to its pro-domain, has been shown to interact with calmodulin, a cytosolic protein displaying high affinity for Ca^2+^ [29, 35]. It was hypothesized that this interaction prevents ADAM10 pro-domain cleavage, and subsequent ADAM10 activation and export to the plasma membrane [35, 36]. The currently accepted model proposes that when cytosolic Ca^2+^ concentration is increased, calmodulin and Ca^2+^ interact, which dissociates calmodulin from pro-ADAM10, as its affinity is lower for the protease. ADAM10 hence becomes available for activation by furin and is eventually ready to process its own substrates [37, 38].

**Fig 5:**
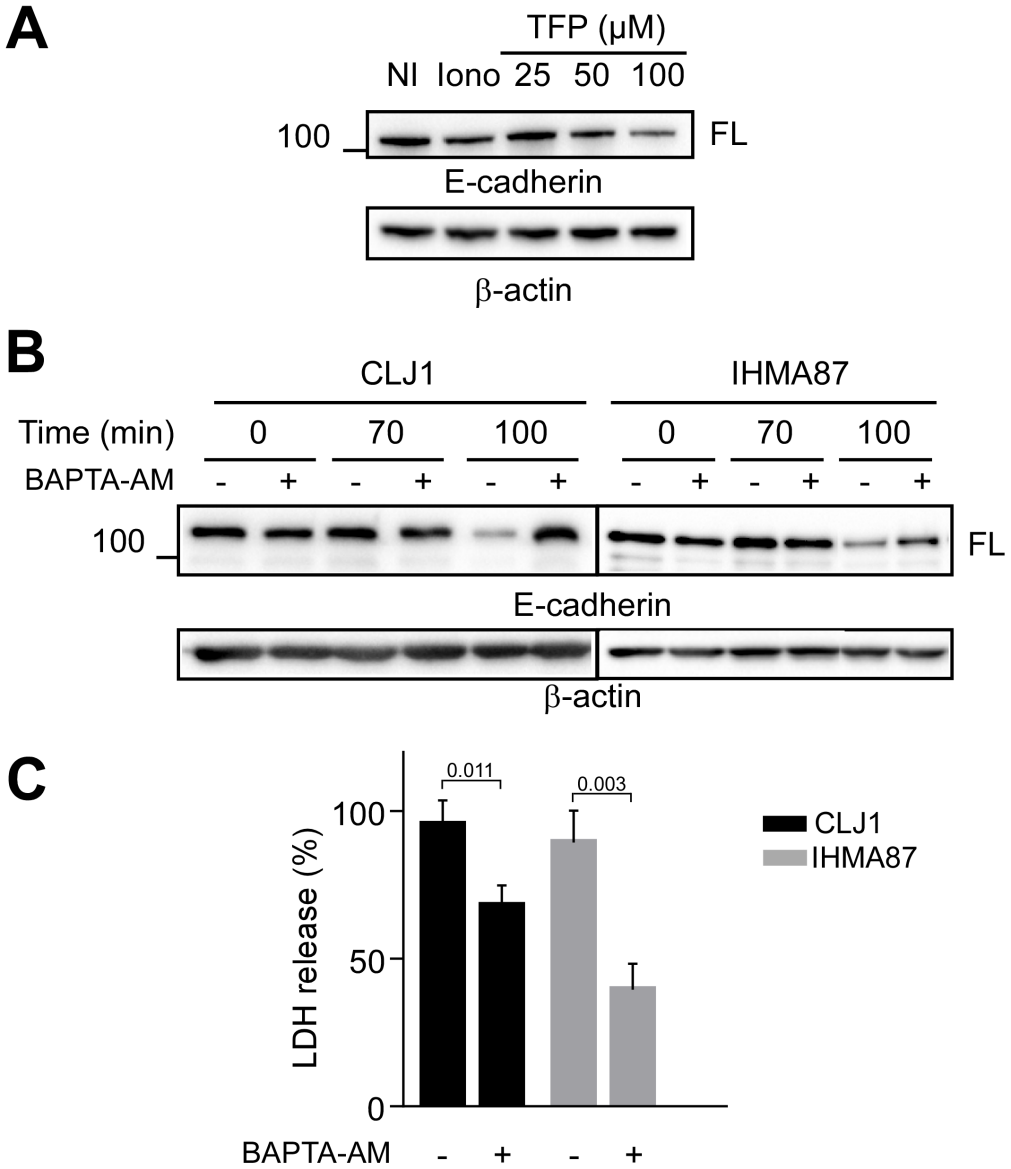
Role of calcium influx in cadherin cleavage and necrosis. **A**. A549 cells were incubated with various concentrations of TFP, as indicated, to impede calmodulin interaction with ADAM10. Ionomycin was used as positive controls. E-cadherin cleavage was assessed by Western blot. The experiment was performed twice. **B**. Western blot analysis of A549 E-cadherin contents after infection with CLJ1 or IHMA87, in presence or absence of BAPTA-AM. Both experiments were performed 3 times. **C**. LDH release of A549 cells infected with either CLJ1 or IHMA87, in presence/ absence of BAPTA-AM. Student’s t-test showed significance between the two treatments for both CLJ1 and IHMA87 data (p-values indicated above the bars). The experiment was performed 3 times.

To test this hypothesis in our system, we first depleted Ca^2+^ in the extracellular medium, but the absence of Ca^2+^ triggered a striking over-secretion of ExlA by the bacteria (S4 Fig) that masked the results on ADAM10 activation. Therefore, we employed a chemical compound, trifluoperazine (TFP), that exhibits high affinity for calmodulin and dissociates calmodulin from its other interactants [29, 35]. TFP treatment of A549 resulted in a dose-dependent induction of E-cadherin cleavage, confirming that calmodulin prevents ADAM10 activation in uninfected A549 cells (Fig 5A).

To further demonstrate the role of intracellular Ca^2+^ in ADAM10 activation, we pre-incubated A549 cells with BAPTA-AM, a cell permeant Ca^2+^ chelator. The chelating action of BAPTA-AM in these cells was tested by Ca^2+^ imaging using Fluo3-AM probe (S5 Fig). Fluo-3 signal inhibition by BAPTA-AM was total in CLJ1-infected cells and almost complete when cells were infected with IHMA87. BAPTA-AM strongly decreased CLJ1-induced E-cadherin cleavage and its action was partial in IHMA87-infected (Fig 5B), consistent with the differential ability of BAPTA-AM to sequester intracellular Ca^2+^ in the two conditions. In conclusion, cytosolic Ca^2+^ is the messenger allowing ADAM10 activation in infected cells.

Interestingly, BAPTA-AM also significantly diminished LDH release induced by CLJ1 or IHMA87 (Fig 5C), suggesting that Ca^2+^ is also involved in ExlA-induced membrane permeability, independently of cadherin cleavage.

### Serratia marcescens *ShlA toxin induces calcium influx*, *ADAM10 activation and cell death*

**Fig 6:**
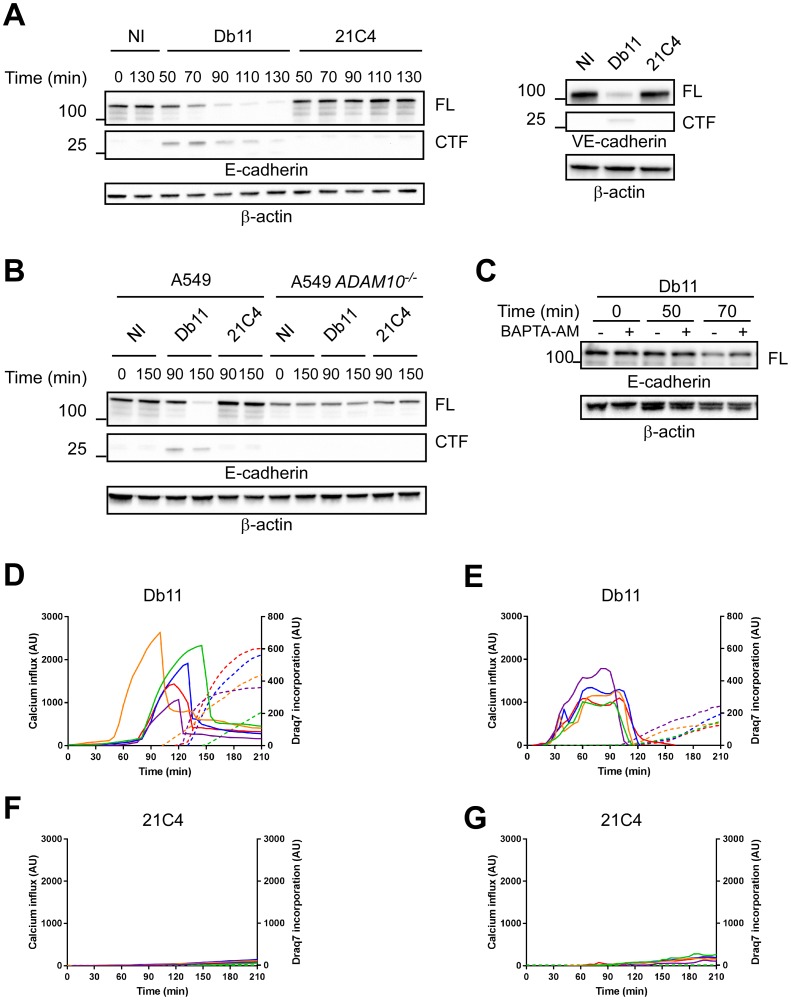
Effects of S. marcescens ShlA on cadherin cleavage and calcium influx. **A**. A549 cells (left) or HUVECs (right) were incubated with the *S*. *marcescens* ShlA-secreting strain Db11, or with the non-ShlA-secreting mutant 21C4. Cellular extracts were analysed for their E- or VE-cadherin contents. The experiment was performed twice for the left panel and once for the right panel. **B**. Similar analysis using A549 *ADAM10*^*-/-*.^ The experiment was performed once. **C**. Similar analysis using A549 cells, in presence/ absence of BAPTA-AM. **D-G**. Intracellular Ca^2+^ contents and plasma membrane permeability were measured using Fluo3-AM and Draq7 fluorescent probes, respectively. A549 cells (**D,F**) and HUVECs (**E,G**) were infected with Db11 (**D,E**) or 21C4 (**F,G**) and fluorescence was recorded on both channels by videomicroscopy. Five cells were analysed in each case; the Fluo3 intensities are represented by straight lines and the Draq7 intensities by dashed lines, using the same colour code for one cell. Data are representative of 8 and 5 independent experiments for A549 and HUVECs, respectively.

As mentioned above, the pore-forming toxin ShlA is homologous to ExlA in terms of sequence identity (35%), secretion pathway and domain organization [12]. We thus examined whether ShlA had the same capacity to promote cadherin cleavage through Ca^2+^ influx and ADAM10 activation. To address this question, we used a ShlA-secreting strain of *S*. *marcescens*, Db11, and its isogenic *shlB* mutant, 21C4, which is impaired in ShlA secretion [39, 40]. Db11 rapidly cleaved E-cadherin with C-terminal cleavage fragment of similar size as ExlA, whereas 21C4 did not induce E-cadherin degradation (Fig 6A left). Similar data were obtained for VE-cadherin (Fig 6A right). Incubation of A549 ADAM10^-/-^ cells with Db11 did not induce E-cadherin cleavage (Fig 6B). E-cadherin degradation was only partially prevented in Db11-infected A549 cells by cell pre-incubation with BAPTA-AM (Fig 6C). However, BAPTA-AM incompletely chelated intracellular Ca^2+^ (S5 Fig). Db11 increased intracellular Ca^2+^ in A549 cells and HUVECs, followed by Draq7 incorporation (Fig 6D and 6E), while no Fluo3 or Draq7 signals were detected with 21C4 (Fig 6F and 6G). Therefore, we conclude that ShlA has the same capacity as ExlA to induce E-cadherin cleavage through Ca^2+^ influx and ADAM10 activation.

## Discussion

Cell-cell junctions of mucosal and vascular barriers play an essential role in impeding the spread of bacterial pathogens in the body (for a review on pore-forming toxin’s action on tissue barriers, see [41]). Here, we employed ExlA- and ShlA-secreting bacteria, and their respective non-secreting mutants, to decipher how the two toxins disrupt adherens junctions.

**Fig 7:**
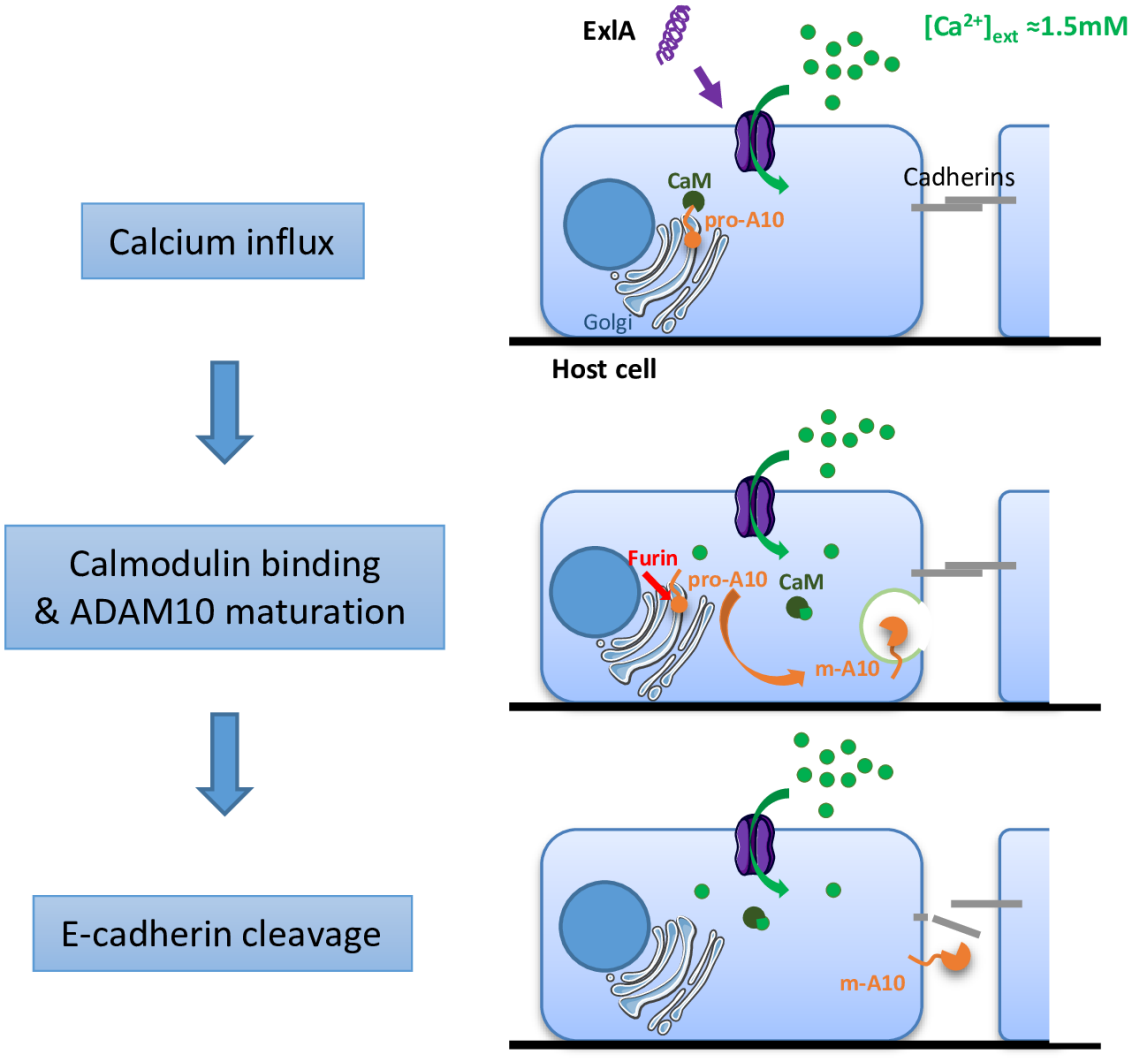
Mechanisms of ExlA/ShlA-induced cadherin cleavage. In uninfected cells, pro-ADAM10 is associated with calmodulin, preventing its maturation and export to the plasma membrane. Pore formation by ExlA or ShlA induces a massive Ca^2+^ influx in host cells. Intracellular Ca^2+^ interacts with the Ca^2+^-binding protein calmodulin, which detaches from pro-ADAM10, allowing its maturation to m-ADAM10. m-ADAM10 cleaves E- and VE-cadherin in epithelial and endothelial cells, respectively, provoking intercellular junction rupture.

Our results demonstrate that one of the primary effects of the cytotoxic action of ExlA and ShlA is that they induce cleavage of E- and VE-cadherins, which disrupts epithelium and endothelium integrity. The massive Ca^2+^ influx triggered by pore formation initiates a cascade of events leading to junction disruption. The model proposed in Fig 7 includes the following steps: in uninfected conditions, calmodulin is associated with pro-ADAM10, preventing its maturation. Calmodulin has a high affinity for Ca^2+^. When Ca^2+^ enters the cell through the ExlA or ShlA pore, it interacts with calmodulin, which presumably alters the conformation of calmodulin and causes its dissociation from ADAM10. Then, the free pro-ADAM10 protein is activated by furin and translocates to the plasma membrane where it cleaves the cadherins. As cadherins are required for cell-cell adhesion, their cleavage is closely followed by cell retraction, owing to actin-cytoskeleton centripetal forces.

Similar data were obtained with CLJ1 and IHMA87, except that the latter was less effective in most assays. The ExlA proteins in these two strains share 99.6% identity, suggesting that the milder effect of IHMA87 may be related to a reduced capacity to secrete ExlA [13].

Ca^2+^ influx started earlier in HUVECs than in A549 cells, suggesting either that the pores are formed more rapidly or that a larger number of pores are formed in HUVECs. This effect could be explained by a higher expression of receptors for ExlA and ShlA at the surface of endothelial cells compared to epithelial cells. However, as no receptor has yet been identified for these two toxins, we cannot currently confirm this hypothesis. For ExlA, the receptor appears to be relatively ubiquitous as ExlA-secreting bacteria are toxic for most tested cell types except erythrocytes [13]. In contrast, ShlA targets erythrocytes, epithelial cells, fibroblasts [17] and endothelial cells (this study), but not myeloid cells. The somewhat different target host cell suggests that the cellular receptors are not identical for the two toxins. Interestingly, the LDH response was unmodified in the absence of ADAM10, when cells were exposed to ExlA- or ShlA-secreting bacteria. Thus, unlike for Hla [31], ADAM10 is not the receptor of either ExlA or ShlA, nor it is involved in ExlA-induced cell death.

Unlike most bacterial pore-forming toxins, ExlA and ShlA are highly unstable in aqueous solution and are biologically inactive when purified. The instability of ExlA and ShlA and the fact that Type IV pili are required for ExlA-related toxicity of the IHMA87 strain [12] suggest that these toxins must be delivered close to host cells where they can rapidly insert into the plasma membrane. The mechanism of insertion into the membrane and the toxin’s oligomerisation capacity remain to be determined.

Because of their role in maintaining tissue barriers, cadherins are frequent targets for bacterial pathogens. Cadherins can be cleaved by ADAM10, which is activated by the calcium imbalance caused by the pore-forming action of the toxins, as previously demonstrated for Hla from *S*. *aureus* and pneumolysin from *Streptococcus pneumoniae* [30, 31, 42] and here for ExlA and ShlA. This mechanism of toxicity might also be used by other pore-forming toxins that are known to promote Ca^2+^ influx, such as streptolysin O (SLO) from *Streptococcus pyogenes*, α-toxin from *Clostridium septicum*, aerolysin from *Aeromonas hydrophila*, and listeriolysin O from *Listeria monocytogenes* [43–46].

Alternatively, E-cadherin can also be cleaved by HtrA bacterial proteases, released by *Helicobacter pylori*, enteropathogenic *E*. *coli*, *Shigella flexneri* and *Campylobacter jejuni [47, 48]*. As mentioned above, VE-cadherin is cleaved by *P*. *aeruginosa* LasB protease [23]. E-cadherin is also the target of *Clostridium botulinum* neurotoxin-A, preventing cadherin homotypic adhesion [49]. Finally, an intermediate mechanism was found for the metalloprotease BFT from *Bacteroides fragilis*, which activates the γ-secretase, promoting E-cadherin cleavage [50]. Thus, bacteria employ several independent but related pathways to target the junctions.

Along the intercellular space, the cluster of E-cadherin molecules forming the adherens junctions, is located closer to the basal side of the cell layer than the tight junctions. The tight junctions seal intercellular gaps on the apical side to restrict the diffusion of molecules (e.g. proteases) present on the apical side of the cell layer. This restriction is less effective in endothelia, where the tight junctions tend to be less developed and where they are mixed with adherens junctions [51]. Bacterial proteases therefore have limited access to E-cadherin in most infection conditions, and the indirect action of pore-forming toxins on E-cadherin appears to be an efficient alternative mechanism to the secretion of proteases directly targeting the junctions.

Because ADAM10 can efficiently cleave adhesive and signalling receptors, its activity is tightly regulated in eukaryotic cells. The subversion mechanism of ADAM10 activation by ExlA and ShlA is probably shared by several pore-forming toxins. This may have functional consequences beyond cellular adhesion because of the numerous substrates of ADAM10, even when cell death is prevented by induction of the membrane repair machinery.

## Methods

### Ethical statement

All protocols in this study were conducted in strict accordance with the French guidelines for the care and use of laboratory animals [52]. The protocols for mouse infection were approved by the animal research committee of the institute (CETEA, project number 13–024) and the French Ministry of Research. Anesthesia was performed using a mixture of xylazine/ketamine and euthanasia by CO2 inhalation.

### P. aeruginosa, S. marcescens *and* E. coli *strains and culture*

The strains and the plasmids used in this study are described in S1 File. Bacteria were grown in liquid LB medium at 37°C with agitation until the cultures reached an optical density value of 1.0 to be in the exponential growth phase. All conditions of infection were performed at MOI of 10, unless indicated in the legend.

### Cell culture

HUVECs were prepared from anonymized human umbilical cords (coming from the Groupe Hospitalier Mutualiste de Grenoble) as previously described [53] and grown in supplemented EBM2 medium (Lonza). A549 (ATCC), A549-E-cadherin GFP and A549 *ADAM10*^-/-^ cells (both generated in this study) were grown in DMEM medium, supplemented with 10% foetal calf serum (all from Life Technologies). Cells were tested for the presence of mycoplasma before freezing vials. Each experiment started from a mycoplasma-free frozen vial. A549 cells were tested for the presence of E-cadherin junctional labelling.

A549 E-cadherin GFP cells were obtained after transfection with pCDNA3.1-E-cadherin-GFP (Addgene) using Lipofectamine 2000 (Life Technologies). Positive cells were selected by antibiotic treatment and fluorescent cells were sorted by MoFlo flow cytometer (Beckman Coulter). When indicated, cells were pretreated 30 min before infection with TFP at indicated concentrations, 0.1 μg/mL phorbol 12-myristate 13-acetate (PMA), 5 μM ionomycin, 10 μg/mL GM6001, 5 μM GI254023X or 25 μM BAPTA-AM (all from Sigma). BAPTA-AM was washed out before the infection step.

For videomicroscopy experiments, cells were seeded at 150,000 cells per well on Labtek II 8-chambered (Thermo Fisher Scientific) coverslips and used 48-h later to obtain highly confluent monolayers. Medium was replaced with non-supplemented EBM-2 medium one hour before infection. Cells were infected and immediately observed by videomicroscopy.

For siRNA experiments, HUVECs were seeded at 5.10^4^ cells/well in P12 plate. The next day, cells were transfected with anti-sense ADAM10 siRNA (UAACAUGACUGGAUAUCUGGG) using Lipofectamine RNAiMAX (Life Technologies). Three days after transfection, cells were analysed by FACScalibur flow cytometer (Becton Dickinson) after staining with ADAM10 antibody (R&D Systems) and anti-mouse-Alexa 488 antibody (Molecular Probes).

### CRISPR/Cas9-based ADAM10 gene knockout

ADAM10 gene knockout was generated by using guide RNA oligos specifying the human *ADAM10* gene (forward: 5’-CACCGGATACCTCTCATATTTACAC; reverse: 5’-AAACGTGTAAATATGAGAGGTATCC). These oligos were designed by using the tool available at http://crispr.mit.edu/. Oligos were obtained from Eurofins Genomics and then cloned into the pSpCas9(BB)-2A-GFP vector (#48138, Addgene). The constructs or the empty vector were transfected into the A549 cells using the Neon Transfection System (Life Technologies). After 24 h, transfected cells were selected by flow cytometry using the GFP signal and were distributed directly in 96-well plates using a BD FACSAria flow cytometer. After 10 days of culture, cells were stained with ADAM10-phycoerythrin antibody (R&D Systems) for 30 min at room temperature. The samples were analysed using a BD Accuri C6 flow cytometer. ADAM10-negative clones were selected and the *ADAM10* gene was sequenced. To further determine if the selected clones had mutations in both alleles, Guide-it sgRNA in vitro Transcription coupled to Guide-it Genotype Confirmation Kits (Clontech Laboratories) were used following the manufacturer recommendations. One clone with both allelic mutations (A549 *ADAM10*^-/-^) was used in this study and confirmed using BD FACScalibur flow cytometer after staining with ADAM10 antibody (R&D systems) and anti-mouse-Alexa488 antibody (Molecular Probes) for 1 h at 4°C for each antibody.

### Mouse pulmonary infection

Pathogen-free BALB/c female mice (8–10 weeks) were obtained from Harlan Laboratories and housed in the institute animal care facility. Bacteria from exponential growth (OD = 1.0) were centrifuged and resuspended in sterile PBS at 0.85x10^8^ per mL. Mice were anesthetized by intraperitoneal administration of a mixture of xylazine (10 mg.Kg^-1^) and ketamine (50 mg.Kg^-1^). Then, 30 μL of bacterial suspension (2.5x10^6^) were deposited into the mouse nostrils. Mice were euthanized by CO_2_ inhalation at 18 h.p.i. and lungs were withdrawn and homogenized in 2 mL of PBS using a Polytron. The number of animals required for the study was deduced from previous work [23]. Randomization was performed by Harlan Laboratories. The experiment did not required blinding.

### Western blotting

Cells and tissues were lysed in Triton X-100 and protein concentration of the lysates was determined with a Micro-BCA kit (Pierce) using BSA as standard. Protein extracts were then separated by SDS-PAGE, transferred onto Hybond ECL membrane (Amersham Biosciences) and revealed with E-cadherin-Cter (BD transduction laboratories), VE-cadherin (Santa Cruz) or β-actin (Sigma) antibodies.

Luminescent signals were revealed using a ChemiDoc (BioRad). Only unsaturated signal intensities are presented.

To examine ExlA secretion, the cell supernatants were collected at 2 h.p.i. and concentrated by trichloroacetic acid (TCA) precipitation. Briefly, 100 μL of 2% Na deoxycholate were added to 10 mL of cellular supernatant and incubated 30 min at 4°C. Then, 1 mL of TCA were added and the mixture was incubated overnight at 4°C. After centrifugation for 15 min, at 15,000 g and 4°C, the pellet was resuspended in 100 μL of electrophoresis loading buffer. The ExlA antibody [3] was used to reveal the western blots.

### Lactate dehydrogenase release

LDH release in the supernatant was measured using the Cytotoxicity Detection Kit from Roche Applied Science, following the recommended protocol. Briefly, cells were seeded at 2.5 10^4^ in 96-well plates two days before, and infected in non-supplemented EBM2. At different post-infection times, 30 μL of supernatant were mixed with 100 μL of reaction mix and OD was read at 492 nm. OD values were subtracted with that of uninfected cells and Triton-solubilized cells were used to determine the total LDH present in the cell culture.

### Confocal videomicroscopy

For fluorescence time-lapse microscopy, cells were imaged using a confocal spinning-disk inverted microscope (Nikon TI-E Eclipse) equipped with an Evolve EMCCD camera. The optical sectioning was performed by a Yokogawa motorized confocal head CSUX1-A1. Images were acquired using an illumination system from Roper Scientific (iLasPulsed) with a CFI Plan Fluor oil immersion objective (40X, N.A. 1.3). Z-series were generated every 10 min using a motorized Z-piezo stage (ASI) by acquiring 25 z-plane images with a step size of 1 μm. Microscope was controlled with MetaMorph software (Molecular Devices). Temperature, CO_2_, and humidity control was performed using a chamlide TC system (TC-A, Quorum technologies). Solid-state 491 and 561 nm lasers (iLas, Roper Scientific) and ET 525/50M (Chroma) and FF01-605/54 (Semrock) emission filters were used for excitation and emission of EGFP and propidium iodide fluorescence, respectively.

### Calcium imaging by wide-field videomicroscopy

Calcium imaging was performed using Fluo3-AM probe (Molecular probes). Cells were seeded at 2.5 10^4^ in Labtek 8-chambered (Thermo Fisher Scientific) one or two days before for A549 cells or HUVECs, respectively. Before experiments, cells were washed twice with PBS. Then cells were loaded with 3 μM Fluo3-AM in PBS or EBM2 containing 2.5 mM probenecid and 40 mg/ml pluronic acid for 1.5 h at room temperature (A549) or 0.5 h at 37°C (HUVECs) in the dark. Cells were washed twice in EBM2. Infection was performed in EBM2 medium with 0.15 μM Draq7 (Abcam). Labtek chambers containing the infected cells were placed in an incubator equilibrated at 37°C and 5% CO2 located on a IX71 Olympus microscope controlled by the CellR Olympus system, automated in x, y, z axis and driven by Xcellence software (Olympus). Fluo3 was excited at 480/40 nm, and emission was collected at 535/50 nm. Draq7 dye was excited at 620/60 nm, and emission was collected at 700/75 nm. Images were captured with a Hammamatsu Orca-ER camera and a 40X (N.A. 1.35) oil objective. Acquisitions were generated every 5 min. The cells used for analysis were chosen at the initial step of recording.

### Statistics

Data on lung cadherin content (Fig 1D) passed the normality test (Shapiro-Wilk's test), but not the equal variance test. Therefore, significance was evaluated on ranks, using a two-sided Mann-Whitney’s test. LDH data (Fig 5C) passed the normality and equal variance tests; significance was thus analysed by a two-sided Student’s t-test. Statistics were performed using SigmaPlot software.

## Supporting information

S1 File Bacterial strains used and supplemental method. (PDF)

S1 Fig Absence of E-cadherin cleavage in cells incubated with an ExoU-positive *P*. *aeruginosa* strain. (PDF)

S2 Fig Absence of pili did not prevent cadherin degradation. (PDF)

S3 Fig ExlA increases bacterial transmigration across A549 monolayers. (PDF)

S4 Fig Calcium depletion increases ExlA secretion. (PDF)

S5 Fig Visualisation of Ca^2+^ titration by BAPTA-AM. (PDF)

## References

[1] Boukerb AM, Marti R, Cournoyer B. Genome Sequences of Three Strains of the Pseudomonas aeruginosa PA7 Clade. Genome Announc. 2015;3(6). doi: 10.1128/genomeA.01366-15 ;. 26586898 10.1128/genomeA.01366-15 PMC4653800

[2] De Soyza A, Perry A, Hall AJ, Sunny SS, Walton KE, Mustafa N, et al Molecular epidemiological analysis suggests cross-infection with Pseudomonas aeruginosa is rare in non-cystic fibrosis bronchiectasis. The European respiratory journal. 2014;43(3):900–3. doi: 10.1183/09031936.00167813 . 24176999 10.1183/09031936.00167813

[3] Elsen S, Huber P, Bouillot S, Coute Y, Fournier P, Dubois Y, et al A type III secretion negative clinical strain of Pseudomonas aeruginosa employs a two-partner secreted exolysin to induce hemorrhagic pneumonia. Cell Host Microbe. 2014;15(2):164–76. doi: 10.1016/j.chom.2014.01.003 . 24528863 10.1016/j.chom.2014.01.003

[4] Huber P, Basso P, Reboud E, Attree I. Pseudomonas aeruginosa renews its virulence factors. Environ Microbiol Rep. 2016 doi: 10.1111/1758-2229.12443 . 27428387 10.1111/1758-2229.12443

[5] King JD, Mulrooney EF, Vinogradov E, Kneidinger B, Mead K, Lam JS. lfnA from Pseudomonas aeruginosa O12 and wbuX from Escherichia coli O145 encode membrane-associated proteins and are required for expression of 2,6-dideoxy-2-acetamidino-L-galactose in lipopolysaccharide O antigen. Journal of bacteriology. 2008;190(5):1671–9. doi: 10.1128/JB.01708-07 ;. 18156256 10.1128/JB.01708-07 PMC2258674

[6] Kos VN, Deraspe M, McLaughlin RE, Whiteaker JD, Roy PH, Alm RA, et al The resistome of Pseudomonas aeruginosa in relationship to phenotypic susceptibility. Antimicrobial agents and chemotherapy. 2015;59(1):427–36. doi: 10.1128/AAC.03954-14 ;. 25367914 10.1128/AAC.03954-14 PMC4291382

[7] Mai-Prochnow A, Bradbury M, Murphy AB. Draft Genome Sequence of Pseudomonas aeruginosa ATCC 9027 (DSM 1128), an Important Rhamnolipid Surfactant Producer and Sterility Testing Strain. Genome Announc. 2015;3(5). doi: 10.1128/genomeA.01259-15 ;. 26514765 10.1128/genomeA.01259-15 PMC4626611

[8] Pirnay JP, Bilocq F, Pot B, Cornelis P, Zizi M, Van Eldere J, et al Pseudomonas aeruginosa population structure revisited. PloS one. 2009;4(11):e7740 doi: 10.1371/journal.pone.0007740 ;. 19936230 10.1371/journal.pone.0007740 PMC2777410

[9] Roy PH, Tetu SG, Larouche A, Elbourne L, Tremblay S, Ren Q, et al Complete genome sequence of the multiresistant taxonomic outlier Pseudomonas aeruginosa PA7. PloS one. 2010;5(1):e8842 doi: 10.1371/journal.pone.0008842 ;. 20107499 10.1371/journal.pone.0008842 PMC2809737

[10] Toska J, Sun Y, Carbonell DA, Foster AN, Jacobs MR, Pearlman E, et al Diversity of Virulence Phenotypes among Type III Secretion Negative Pseudomonas aeruginosa Clinical Isolates. PloS one. 2014;9(1):e86829 doi: 10.1371/journal.pone.0086829 . 24466261 10.1371/journal.pone.0086829 PMC3900666

[11] van Belkum A, Soriaga LB, LaFave MC, Akella S, Veyrieras JB, Barbu EM, et al Phylogenetic Distribution of CRISPR-Cas Systems in Antibiotic-Resistant Pseudomonas aeruginosa. MBio. 2015;6(6):e01796–15. doi: 10.1128/mBio.01796-15 ;. 26604259 10.1128/mBio.01796-15 PMC4669384

[12] Basso P, Ragno M, Elsen S, Reboud E, Golovkine G, Bouillot S, et al Pseudomonas aeruginosa Pore-Forming Exolysin and Type IV Pili Cooperate To Induce Host Cell Lysis. MBio. 2017;8(1). doi: 10.1128/mBio.02250-16 ;. 28119472 10.1128/mBio.02250-16 PMC5263249

[13] Reboud E, Elsen S, Bouillot S, Golovkine G, Basso P, Jeannot K, et al Phenotype and toxicity of the recently discovered exlA-positive Pseudomonas aeruginosa strains collected worldwide. Environ Microbiol. 2016;18(10):3425–39. doi: 10.1111/1462-2920.13262 . 26914644 10.1111/1462-2920.13262

[14] Hertle R. The family of Serratia type pore forming toxins. Current protein & peptide science. 2005;6(4):313–25. . 16101433 10.2174/1389203054546370

[15] Goldstein JD, Godleski JJ, Balikian JP, Herman PG. Pathologic patterns of Serratia marcescens pneumonia. Hum Pathol. 1982;13(5):479–84. . 7042532 10.1016/s0046-8177(82)80031-2

[16] Gonzalez-Juarbe N, Mares CA, Hinojosa CA, Medina JL, Cantwell A, Dube PH, et al Requirement for Serratia marcescens cytolysin in a murine model of hemorrhagic pneumonia. Infection and immunity. 2015;83(2):614–24. doi: 10.1128/IAI.01822-14 ;. 25422267 10.1128/IAI.01822-14 PMC4294263

[17] Hertle R, Hilger M, Weingardt-Kocher S, Walev I. Cytotoxic action of Serratia marcescens hemolysin on human epithelial cells. Infection and immunity. 1999;67(2):817–25. ;. 9916096 10.1128/iai.67.2.817-825.1999 PMC96392

[18] Brillard J, Duchaud E, Boemare N, Kunst F, Givaudan A. The PhlA hemolysin from the entomopathogenic bacterium Photorhabdus luminescens belongs to the two-partner secretion family of hemolysins. Journal of bacteriology. 2002;184(14):3871–8. doi: 10.1128/JB.184.14.3871-3878.2002 ;. 12081958 10.1128/JB.184.14.3871-3878.2002 PMC135187

[19] Brumbach KC, Eason BD, Anderson LK. The Serratia-type hemolysin of Chromobacterium violaceum. FEMS microbiology letters. 2007;267(2):243–50. doi: 10.1111/j.1574-6968.2006.00566.x . 17169000 10.1111/j.1574-6968.2006.00566.x

[20] Hirono I, Tange N, Aoki T. Iron-regulated haemolysin gene from Edwardsiella tarda. Molecular microbiology. 1997;24(4):851–6. . 9194711 10.1046/j.1365-2958.1997.3971760.x

[21] Palmer KL, Munson RS Jr. Cloning and characterization of the genes encoding the hemolysin of Haemophilus ducreyi. Molecular microbiology. 1995;18(5):821–30. . 8825086 10.1111/j.1365-2958.1995.18050821.x

[22] Uphoff TS, Welch RA. Nucleotide sequencing of the Proteus mirabilis calcium-independent hemolysin genes (hpmA and hpmB) reveals sequence similarity with the Serratia marcescens hemolysin genes (shlA and shlB). Journal of bacteriology. 1990;172(3):1206–16. ;. 2407716 10.1128/jb.172.3.1206-1216.1990 PMC208585

[23] Golovkine G, Faudry E, Bouillot S, Voulhoux R, Attree I, Huber P. VE-cadherin cleavage by LasB protease from Pseudomonas aeruginosa facilitates type III secretion system toxicity in endothelial cells. PLoS pathogens. 2014;10(3):e1003939 doi: 10.1371/journal.ppat.1003939 ;. 24626230 10.1371/journal.ppat.1003939 PMC3953407

[24] Hauser AR. The type III secretion system of Pseudomonas aeruginosa: infection by injection. Nature reviews. 2009;7(9):654–65. doi: 10.1038/nrmicro2199 . 19680249 10.1038/nrmicro2199 PMC2766515

[25] Dreymueller D, Pruessmeyer J, Groth E, Ludwig A. The role of ADAM-mediated shedding in vascular biology. European journal of cell biology. 2012;91(6–7):472–85. doi: 10.1016/j.ejcb.2011.09.003 . 22138087 10.1016/j.ejcb.2011.09.003

[26] Maretzky T, Reiss K, Ludwig A, Buchholz J, Scholz F, Proksch E, et al ADAM10 mediates E-cadherin shedding and regulates epithelial cell-cell adhesion, migration, and beta-catenin translocation. Proceedings of the National Academy of Sciences of the United States of America. 2005;102(26):9182–7. doi: 10.1073/pnas.0500918102 ;. 15958533 10.1073/pnas.0500918102 PMC1166595

[27] Pruessmeyer J, Ludwig A. The good, the bad and the ugly substrates for ADAM10 and ADAM17 in brain pathology, inflammation and cancer. Semin Cell Dev Biol. 2009;20(2):164–74. doi: 10.1016/j.semcdb.2008.09.005 . 18951988 10.1016/j.semcdb.2008.09.005

[28] Schulz B, Pruessmeyer J, Maretzky T, Ludwig A, Blobel CP, Saftig P, et al ADAM10 regulates endothelial permeability and T-Cell transmigration by proteolysis of vascular endothelial cadherin. Circulation research. 2008;102(10):1192–201. doi: 10.1161/CIRCRESAHA.107.169805 . 18420943 10.1161/CIRCRESAHA.107.169805 PMC2818019

[29] Horiuchi K, Le Gall S, Schulte M, Yamaguchi T, Reiss K, Murphy G, et al Substrate selectivity of epidermal growth factor-receptor ligand sheddases and their regulation by phorbol esters and calcium influx. Molecular biology of the cell. 2007;18(1):176–88. doi: 10.1091/mbc.E06-01-0014 ;. 17079736 10.1091/mbc.E06-01-0014 PMC1751309

[30] Powers ME, Kim HK, Wang Y, Bubeck Wardenburg J. ADAM10 mediates vascular injury induced by Staphylococcus aureus alpha-hemolysin. The Journal of infectious diseases. 2012;206(3):352–6. doi: 10.1093/infdis/jis192 ;. 22474035 10.1093/infdis/jis192 PMC3392186

[31] Wilke GA, Bubeck Wardenburg J. Role of a disintegrin and metalloprotease 10 in Staphylococcus aureus alpha-hemolysin-mediated cellular injury. Proceedings of the National Academy of Sciences of the United States of America. 2010;107(30):13473–8. doi: 10.1073/pnas.1001815107 ;. 20624979 10.1073/pnas.1001815107 PMC2922128

[32] Ludwig A, Hundhausen C, Lambert MH, Broadway N, Andrews RC, Bickett DM, et al Metalloproteinase inhibitors for the disintegrin-like metalloproteinases ADAM10 and ADAM17 that differentially block constitutive and phorbol ester-inducible shedding of cell surface molecules. Comb Chem High Throughput Screen. 2005;8(2):161–71. . 15777180 10.2174/1386207053258488

[33] Marambaud P, Shioi J, Serban G, Georgakopoulos A, Sarner S, Nagy V, et al A presenilin-1/gamma-secretase cleavage releases the E-cadherin intracellular domain and regulates disassembly of adherens junctions. The EMBO journal. 2002;21(8):1948–56. doi: 10.1093/emboj/21.8.1948 ;. 11953314 10.1093/emboj/21.8.1948 PMC125968

[34] Jacob T, Lee RJ, Engel JN, Machen TE. Modulation of cytosolic Ca(2+) concentration in airway epithelial cells by Pseudomonas aeruginosa. Infection and immunity. 2002;70(11):6399–408. doi: 10.1128/IAI.70.11.6399-6408.2002 ;. 12379720 10.1128/IAI.70.11.6399-6408.2002 PMC130342

[35] Nagano O, Murakami D, Hartmann D, De Strooper B, Saftig P, Iwatsubo T, et al Cell-matrix interaction via CD44 is independently regulated by different metalloproteinases activated in response to extracellular Ca(2+) influx and PKC activation. The Journal of cell biology. 2004;165(6):893–902. doi: 10.1083/jcb.200310024 ;. 15197174 10.1083/jcb.200310024 PMC2172408

[36] Ponnuchamy B, Khalil RA. Role of ADAMs in endothelial cell permeability: cadherin shedding and leukocyte rolling. Circulation research. 2008;102(10):1139–42. doi: 10.1161/CIRCRESAHA.108.177394 ;. 18497310 10.1161/CIRCRESAHA.108.177394 PMC2746645

[37] Ebsen H, Lettau M, Kabelitz D, Janssen O. Identification of SH3 domain proteins interacting with the cytoplasmic tail of the a disintegrin and metalloprotease 10 (ADAM10). PloS one. 2014;9(7):e102899 doi: 10.1371/journal.pone.0102899 ;. 25036101 10.1371/journal.pone.0102899 PMC4103893

[38] Seals DF, Courtneidge SA. The ADAMs family of metalloproteases: multidomain proteins with multiple functions. Genes & development. 2003;17(1):7–30. doi: 10.1101/gad.1039703 . 12514095 10.1101/gad.1039703

[39] Flyg C, Kenne K, Boman HG. Insect pathogenic properties of Serratia marcescens: phage-resistant mutants with a decreased resistance to Cecropia immunity and a decreased virulence to Drosophila. J Gen Microbiol. 1980;120(1):173–81. doi: 10.1099/00221287-120-1-173 . 7012273 10.1099/00221287-120-1-173

[40] Kurz CL, Chauvet S, Andres E, Aurouze M, Vallet I, Michel GP, et al Virulence factors of the human opportunistic pathogen Serratia marcescens identified by in vivo screening. The EMBO journal. 2003;22(7):1451–60. doi: 10.1093/emboj/cdg159 ;. 12660152 10.1093/emboj/cdg159 PMC152903

[41] Los FC, Randis TM, Aroian RV, Ratner AJ. Role of pore-forming toxins in bacterial infectious diseases. Microbiol Mol Biol Rev. 2013;77(2):173–207. doi: 10.1128/MMBR.00052-12 ;. 23699254 10.1128/MMBR.00052-12 PMC3668673

[42] Inoshima I, Inoshima N, Wilke GA, Powers ME, Frank KM, Wang Y, et al A Staphylococcus aureus pore-forming toxin subverts the activity of ADAM10 to cause lethal infection in mice. Nature medicine. 2011;17(10):1310–4. doi: 10.1038/nm.2451 . 21926978 10.1038/nm.2451 PMC3192248

[43] Cywes Bentley C, Hakansson A, Christianson J, Wessels MR. Extracellular group A Streptococcus induces keratinocyte apoptosis by dysregulating calcium signalling. Cellular microbiology. 2005;7(7):945–55. doi: 10.1111/j.1462-5822.2005.00525.x . 15953027 10.1111/j.1462-5822.2005.00525.x

[44] Gekara NO, Groebe L, Viegas N, Weiss S. Listeria monocytogenes desensitizes immune cells to subsequent Ca2+ signaling via listeriolysin O-induced depletion of intracellular Ca2+ stores. Infection and immunity. 2008;76(2):857–62. doi: 10.1128/IAI.00622-07 ;. 18056478 10.1128/IAI.00622-07 PMC2223449

[45] Kennedy CL, Smith DJ, Lyras D, Chakravorty A, Rood JI. Programmed cellular necrosis mediated by the pore-forming alpha-toxin from Clostridium septicum. PLoS pathogens. 2009;5(7):e1000516 doi: 10.1371/journal.ppat.1000516 ;. 19609357 10.1371/journal.ppat.1000516 PMC2705182

[46] Nelson KL, Brodsky RA, Buckley JT. Channels formed by subnanomolar concentrations of the toxin aerolysin trigger apoptosis of T lymphomas. Cellular microbiology. 1999;1(1):69–74. . 11207542 10.1046/j.1462-5822.1999.00009.x

[47] Hoy B, Geppert T, Boehm M, Reisen F, Plattner P, Gadermaier G, et al Distinct roles of secreted HtrA proteases from gram-negative pathogens in cleaving the junctional protein and tumor suppressor E-cadherin. The Journal of biological chemistry. 2012;287(13):10115–20. doi: 10.1074/jbc.C111.333419 ;. 22337879 10.1074/jbc.C111.333419 PMC3323053

[48] Hoy B, Lower M, Weydig C, Carra G, Tegtmeyer N, Geppert T, et al Helicobacter pylori HtrA is a new secreted virulence factor that cleaves E-cadherin to disrupt intercellular adhesion. EMBO Rep. 2010;11(10):798–804. doi: 10.1038/embor.2010.114 ;. 20814423 10.1038/embor.2010.114 PMC2948180

[49] Lee K, Zhong X, Gu S, Kruel AM, Dorner MB, Perry K, et al Molecular basis for disruption of E-cadherin adhesion by botulinum neurotoxin A complex. Science (New York, NY. 2014;344(6190):1405–10. doi: 10.1126/science.1253823 ;. 24948737 10.1126/science.1253823 PMC4164303

[50] Wu S, Rhee KJ, Zhang M, Franco A, Sears CL. Bacteroides fragilis toxin stimulates intestinal epithelial cell shedding and gamma-secretase-dependent E-cadherin cleavage. Journal of cell science. 2007;120(Pt 11):1944–52. doi: 10.1242/jcs.03455 ;. 17504810 10.1242/jcs.03455 PMC3056613

[51] Wallez Y, Huber P. Endothelial adherens and tight junctions in vascular homeostasis, inflammation and angiogenesis. Biochimica et biophysica acta. 2008;1778(3):794–809. doi: 10.1016/j.bbamem.2007.09.003 . 17961505 10.1016/j.bbamem.2007.09.003

[52] Council NR. Guide for the Care and Use of Laboratory Animals—French Version. Washington, DC: The National Academies Press; 1996 134 p. 25121211

[53] Huber P, Bouillot S, Elsen S, Attree I. Sequential inactivation of Rho GTPases and Lim kinase by Pseudomonas aeruginosa toxins ExoS and ExoT leads to endothelial monolayer breakdown. Cell Mol Life Sci. 2014;71(10):1927–41. doi: 10.1007/s00018-013-1451-9 . 23974244 10.1007/s00018-013-1451-9 PMC11113219

