# Supplementary material for: Pseudomonas aeruginosa ExlA and Serratia marcescens ShlA trigger cadherin cleavage by promoting calcium influx and ADAM10 activation
Source: PLoS Pathog. 2017 Aug 23;13(8):e1006579. doi: 10.1371/journal.ppat.1006579 (PMC5584975; doi:10.1371/journal.ppat.1006579)
Supplement: S1 File — (PDF) [file ppat.1006579.s001.pdf]

Pseudomonas aeruginosa ExlA and *Serratia marcescens* ShlA trigger cadherin cleavage by promoting calcium influx and ADAM10 activation

Emeline Reboud[1], Stéphanie Bouillot[1], Sabine Patot[2], Benoît Béganton[1], Ina Attrée[1] and Philippe

Huber[1*]

Supporting Information

S1 Table: *P. aeruginosa*, *S. marcescens* and *E. coli* strains used in this work

Strains	Description	Abbreviations	Refs
Pseudomonas aeruginosa			
PAO1F (RP1831)	Wild-type laboratory strain T3SS+ (*exoS,T,Y*)	PAO1F	[1]
CHA	Wild-type laboratory strain T3SS+ (*exoS,T,Y*)	CHA	[2]
PA14	Wild-type laboratory strain T3SS+ (*exoU,T,Y*)	PA14	[3]
CLJ1	Clinical strain T3SS-, ExlA+	CLJ1	[4]
IHMA879472	Clinical strain T3SS-, ExlA+	IHMA87	[5]
IHMA87Δ*exlA*	IHMA87 with *exlA* inactivated	IHMA87Δ*exlA*	[6]
IHMA87Δ*exlA/exlA*	IHMA87Δ*exlA* with plasmidic expression of *exlA*	IHMA87Δ*exlA/exlA*	This work
IHMA87Δ*exlA::exlBA*	IHMA87Δ*exlA* with chromosomal insertion of *exlBA*	IHMA87Δ*exlA::exlBA*	[6]
IHMA87Δ*pilA*	IHMA87 with *pilA* deletion	IHMA87Δ*pilA*	[6]
Serratia marcescens			
Db11	Clinical strain expressing ShlA		*[7]*
21C4	*shlB* mutant of Db11 with no secretion of ShlA		[8]
Escherichia coli			
TOP10 pSW196	Strain containing pSW196 vector only	*E. coli*-vector	[4]
TOP10 pSW196exlBA	Strain expressing the *exlBA* operon	*E. coli-exlBA*	[4]

References

1. Cisz M, Lee PC, Rietsch A (2008) ExoS controls the cell contact-mediated switch to effector secretion in Pseudomonas aeruginosa. J Bacteriol 190: 2726-2738.
2. Toussaint B, Delic-Attree I, Vignais PM (1993) Pseudomonas aeruginosa contains an IHF-like protein that binds to the algD promoter. Biochem Biophys Res Commun 196: 416-421.
3. Berthelot P, Attree I, Plesiat P, Chabert J, de Bentzmann S, et al. (2003) Genotypic and phenotypic analysis of type III secretion system in a cohort of Pseudomonas aeruginosa bacteremia

isolates: evidence for a possible association between O serotypes and exo genes. J Infect Dis 188: 512-518.
4. Elsen S, Huber P, Bouillot S, Coute Y, Fournier P, et al. (2014) A type III secretion negative clinical strain of Pseudomonas aeruginosa employs a two-partner secreted exolysin to induce hemorrhagic pneumonia. Cell Host Microbe 15: 164-176.
5. Kos VN, Deraspe M, McLaughlin RE, Whiteaker JD, Roy PH, et al. (2015) The resistome of Pseudomonas aeruginosa in relationship to phenotypic susceptibility. Antimicrob Agents Chemother 59: 427-436.
6. Basso P, Ragno M, Elsen S, Reboud E, Golovkine G, et al. (2017) Pseudomonas aeruginosa Pore-Forming Exolysin and Type IV Pili Cooperate To Induce Host Cell Lysis. MBio 8.
7. Flyg C, Kenne K, Boman HG (1980) Insect pathogenic properties of Serratia marcescens: phage-resistant mutants with a decreased resistance to Cecropia immunity and a decreased virulence to Drosophila. J Gen Microbiol 120: 173-181.
8. Kurz CL, Chauvet S, Andres E, Aurouze M, Vallet I, et al. (2003) Virulence factors of the human opportunistic pathogen Serratia marcescens identified by in vivo screening. EMBO J 22: 1451-1460.

Supplemental Method

Bacterial transmigration across Transwell devices

Three days before infection, A549 cells ($6\ 10^4$) were seeded in DMEM medium on Transwell filters (Greiner ThinCert 24 well, 3.0 µm). At day 0, the medium was replaced and bacteria (MOI 10) were added in the upper compartment. At 4 h.p.i., 100 µL of medium in the lower compartment were withdrawn, serially diluted and spotted onto LB-agar plates to determine the number of transmigrated bacteria.
