# Supplementary material for: Pseudomonas aeruginosa ExlA and Serratia marcescens ShlA trigger cadherin cleavage by promoting calcium influx and ADAM10 activation
Source: PLoS Pathog. 2017 Aug 23;13(8):e1006579. doi: 10.1371/journal.ppat.1006579 (PMC5584975; doi:10.1371/journal.ppat.1006579)
Supplement: S1 Fig — (PDF) [file ppat.1006579.s002.pdf]

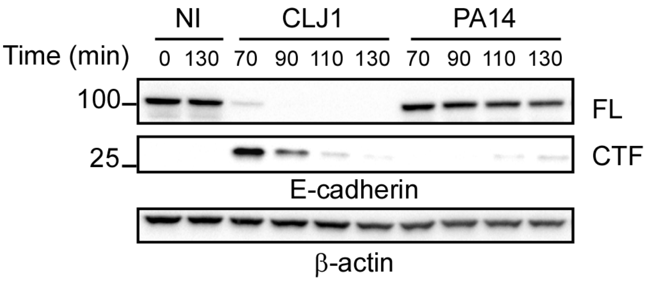

S1 Figure: Absence of E-cadherin cleavage in cells incubated with an ExoU-positive *P. aeruginosa* strain.

A549 cells were incubated with CLJ1, PA14 (ExoU+) or were mock-infected with LB. Cellular extracts were analysed for their E-cadherin contents. The experiment was performed twice with similar results.
