# Supplementary material for: Pseudomonas aeruginosa ExlA and Serratia marcescens ShlA trigger cadherin cleavage by promoting calcium influx and ADAM10 activation
Source: PLoS Pathog. 2017 Aug 23;13(8):e1006579. doi: 10.1371/journal.ppat.1006579 (PMC5584975; doi:10.1371/journal.ppat.1006579)
Supplement: S2 Fig — (PDF) [file ppat.1006579.s003.pdf]

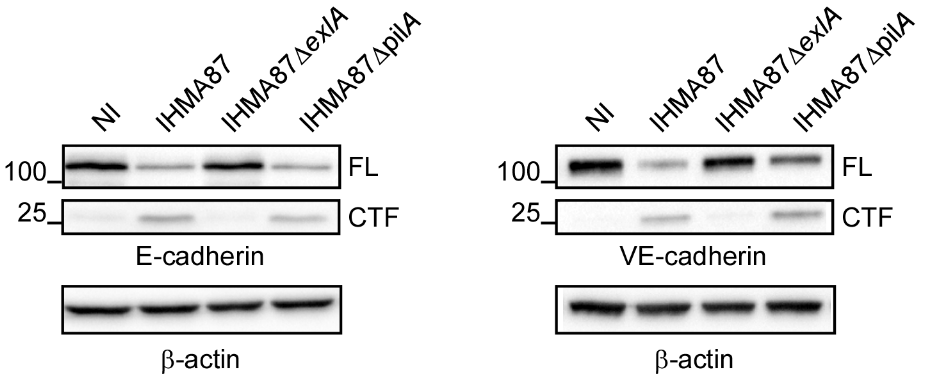

S2 Figure: Absence of pili did not prevent cadherin degradation

A549 cells (left) or HUVECs (right) were incubated with IHMA87, IHMA87Δ*exlA* or IHMA87Δ*pilA* bacteria. Cellular extracts were analysed for their E- or VE-cadherin contents after 2 h.p.i.. The results are representative of three experiments for A549 cells and two experiments for HUVECs.
