# Supplementary material for: Pseudomonas aeruginosa ExlA and Serratia marcescens ShlA trigger cadherin cleavage by promoting calcium influx and ADAM10 activation
Source: PLoS Pathog. 2017 Aug 23;13(8):e1006579. doi: 10.1371/journal.ppat.1006579 (PMC5584975; doi:10.1371/journal.ppat.1006579)
Supplement: S3 Fig — (PDF) [file ppat.1006579.s004.pdf]

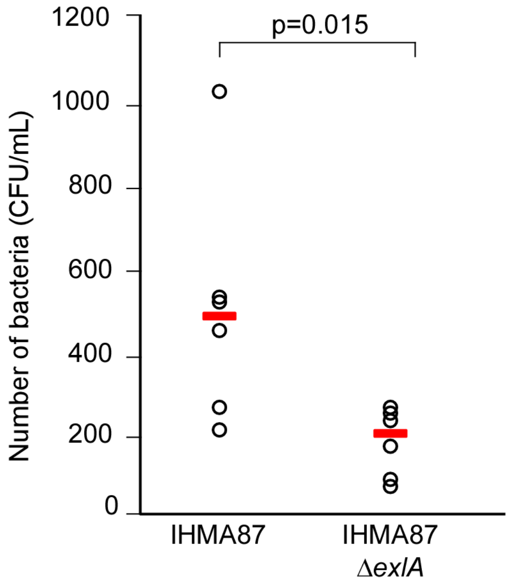

S3 Figure: ExlA increases bacterial transmigration across A549 monolayers

A549 cells were seeded onto Transwell filters and grown until confluency. IHMA87 or IHMA87Δ*exlA* bacteria were added to the upper compartment and bacteria were collected at 4 h.p.i. in the lower compartment. CFU were counted by plating onto LB plates. The graph represents individual triplicates from two independent experiments. The red bars show the medians. Significance was calculated using Mann-Whitney's test.
