# Supplementary material for: Pseudomonas aeruginosa ExlA and Serratia marcescens ShlA trigger cadherin cleavage by promoting calcium influx and ADAM10 activation
Source: PLoS Pathog. 2017 Aug 23;13(8):e1006579. doi: 10.1371/journal.ppat.1006579 (PMC5584975; doi:10.1371/journal.ppat.1006579)
Supplement: S4 Fig — (PDF) [file ppat.1006579.s005.pdf]

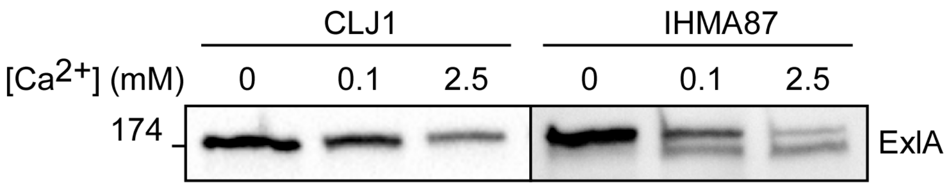

S4 Figure: Calcium depletion increases ExlA secretion

A549 cells were incubated with CLJ1 or IHMA87 bacteria in Tyrode buffer with various Ca^{2+} concentrations. The supernatants were harvested after 2 hours, TCA-precipitated and analysed by Western blot for their content in ExlA. The results are representative of three experiments.
