# Supplementary material for: Pseudomonas aeruginosa ExlA and Serratia marcescens ShlA trigger cadherin cleavage by promoting calcium influx and ADAM10 activation
Source: PLoS Pathog. 2017 Aug 23;13(8):e1006579. doi: 10.1371/journal.ppat.1006579 (PMC5584975; doi:10.1371/journal.ppat.1006579)
Supplement: S5 Fig — (PDF) [file ppat.1006579.s006.pdf]

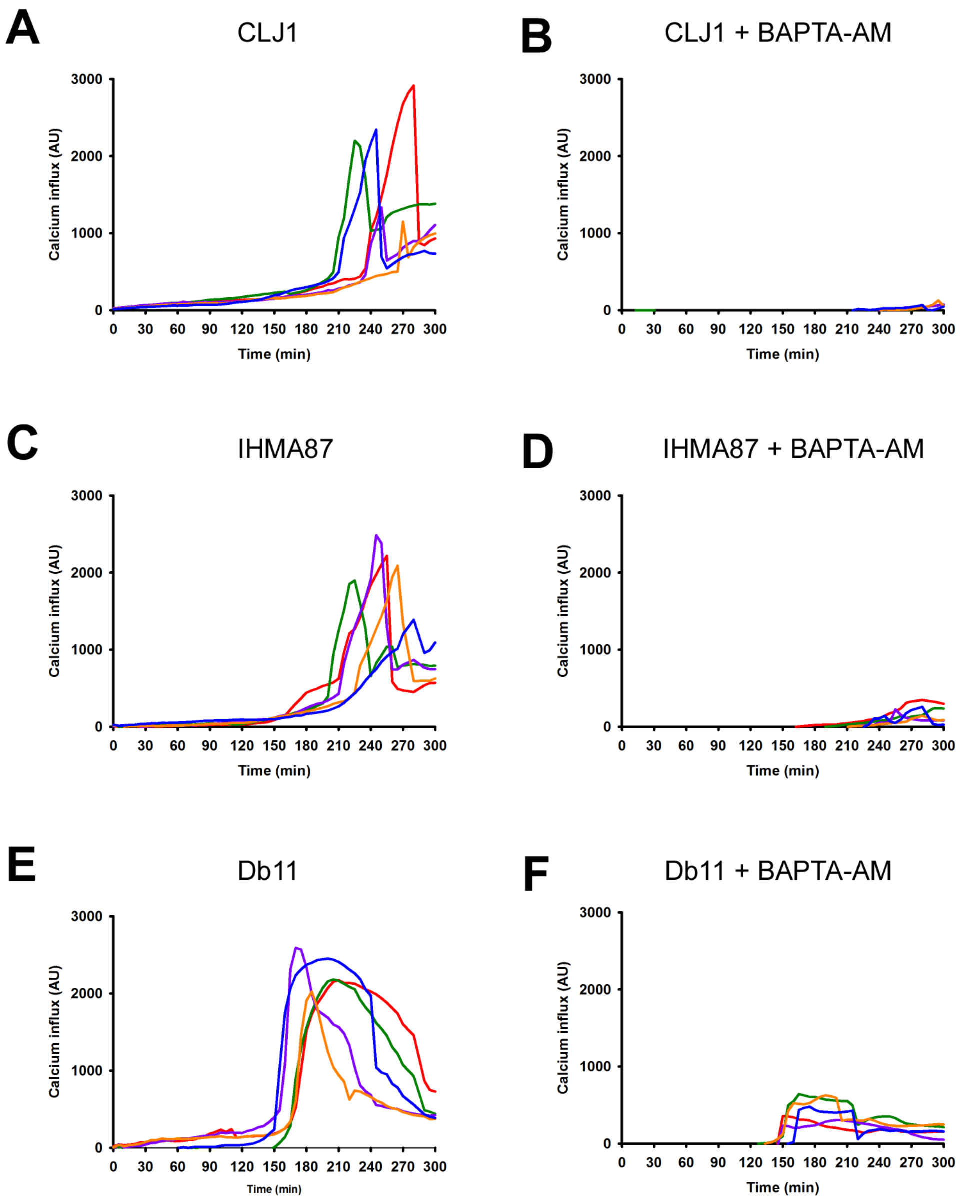

S5 Figure: Visualisation of Ca^{2+} titration by BAPTA-AM

A549 cells were pre-incubated with BAPTA-AM and Fluo3-AM and incubated with CLJ1, IHMA87 or Db11. Fluo3 signals were followed by videomicroscopy. Each coloured line represent the signal for a single cell. BAPTA-AM totally or partially inhibited the available cytosolic Ca^{2+} depending on the strain. The data are representative of three independent experiments.
